# Nrf2 Activation Provides Atheroprotection in Diabetic Mice Through Concerted Upregulation of Antioxidant, Anti-inflammatory, and Autophagy Mechanisms

**DOI:** 10.3389/fphar.2018.00819

**Published:** 2018-07-31

**Authors:** Iolanda Lazaro, Laura Lopez-Sanz, Susana Bernal, Ainhoa Oguiza, Carlota Recio, Ana Melgar, Luna Jimenez-Castilla, Jesus Egido, Julio Madrigal-Matute, Carmen Gomez-Guerrero

**Affiliations:** ^1^Renal, Vascular and Diabetes Research Lab, IIS-Fundacion Jimenez Diaz, Autonoma University of Madrid, Madrid, Spain; ^2^Spanish Biomedical Research Centre in Diabetes and Associated Metabolic Disorders, Madrid, Spain; ^3^Department of Developmental and Molecular Biology, Institute for Aging Studies, Albert Einstein College of Medicine, New York City, NY, United States

**Keywords:** nuclear factor (erythroid-derived 2)-like 2, redox balance, autophagy, inflammation, diabetes complications

## Abstract

Interactive relationships between metabolism, inflammation, oxidative stress, and autophagy in the vascular system play a key role in the pathogenesis of diabetic cardiovascular disease. Nuclear factor (erythroid-derived 2)-like 2 (Nrf2) is a stress-sensitive guarantor of cellular homeostasis, which cytoprotective contributions extend beyond the antioxidant defense. We investigated the beneficial effects and underlying mechanisms of the Nrf2 inducer tert-butyl hydroquinone (tBHQ) on diabetes-driven atherosclerosis. In the experimental model of streptozotocin-induced diabetes in apolipoprotein E-deficient mice, treatment with tBHQ increased Nrf2 activity in macrophages and vascular smooth muscle cells within atherosclerotic lesions. Moreover, tBHQ significantly decreased the size, extension and lipid content of atheroma plaques, and attenuated inflammation by reducing lesional macrophages (total number and M1/M2 phenotype balance), foam cell size and chemokine expression. Atheroprotection was accompanied by both systemic and local antioxidant effects, characterized by lower levels of superoxide anion and oxidative DNA marker 8-hydroxy-2′-deoxyguanosine, reduced expression of NADPH oxidase subunits, and increased antioxidant capacity. Interestingly, tBHQ treatment upregulated the gene and protein expression of autophagy-related molecules and also enhanced autophagic flux in diabetic mouse aorta. *In vitro*, Nrf2 activation by tBHQ suppressed cytokine-induced expression of pro-inflammatory and oxidative stress genes, altered macrophage phenotypes, and promoted autophagic activity. Our results reinforce pharmacological Nrf2 activation as a promising atheroprotective approach in diabetes, according to the plethora of cytoprotective mechanisms involved in the resolution of inflammation and oxidative stress, and restoring autophagy.

## Introduction

Diabetes is a major risk factor for atherosclerosis (Eckel et al., 2002). Vascular complications are the leading cause of disability and mortality in diabetic patients (Rask-Madsen and King, 2013). Chronic hyperglycemia has been linked to a low-grade inflammatory state, in which excessive production of pro-inflammatory cytokines and ROS by mitochondria and NADPH oxidase (Nox), and impaired antioxidant and autophagic mechanisms contribute to the pathology and complications of diabetes (Jung et al., 2008; Rask-Madsen and King, 2013). Therefore, in addition to intensive glycemic control, there is an urgent need for novel therapies to limit vascular inflammation and restore redox balance in diabetic patients.

Nuclear factor (erythroid-derived 2)-like 2 (Nrf2) is a redox-sensitive transcription factor and a master regulator of cytoprotective and antioxidant genes including heme oxygenase 1 (HMOX1), superoxide dismutase 1 (SOD1) and catalase (Itoh et al., 1997). Beyond the resolution of oxidative stress, additional roles of Nrf2 include the inhibition of inflammation by directly impeding pro-inflammatory cytokine gene transcription (Kobayashi et al., 2016), the regulation of genes involved in lipid metabolism, apoptosis and cell death, and the induction of autophagy markers such as sequestosome 1 (SQTSM1/p62) and autophagy-related protein 5 (ATG5) (Pajares et al., 2016). Nrf2 under the canonical pathway remains bound to Kelch-like ECH-associated protein 1 (KEAP1) which facilitates the ubiquitination and constant proteasomal degradation of Nrf2 (Itoh et al., 1997; Ma et al., 2011). Upon cellular stress, KEAP1 inhibitory effect is abolished, and subsequently stabilized Nrf2 translocates into the nucleus and binds to the AREs to activate transcription of detoxifying and antioxidant genes (Itoh et al., 1997). Furthermore, the non-canonical pathway tightly links Nrf2 and autophagy. In this cysteine-independent mechanism, SQSTM1/p62 sequesters KEAP1 to autophagic degradation that ultimately leads to Nrf2 stabilization and transactivation of Nrf2-dependent genes (Jain et al., 2010; Komatsu et al., 2010; Lau et al., 2010). Autophagy is a highly conserved lysosomal degradation pathway that removes protein aggregates and damaged organelles to maintain metabolic processes and cellular homeostasis (Singh et al., 2009; Levine and Klionsky, 2017).

Nrf2-mediated pathway is increasingly proposed as a way to prevent or treat disease. In preclinical models, pharmacological Nrf2 activators including 1,2-mercapto-3-sulfur ketone derivatives (oltipraz), isopropyl sulfur cyanogen compounds (sulforaphane), selenium-containing drugs (ebselen), natural products (resveratrol and curcumin) and phenolic compounds tBHQ, have been used as treatments for cancer, cardiovascular, metabolic and neurodegenerative diseases (Jiang et al., 2010; Ma, 2013; Tan and de Haan, 2014; Tan et al., 2014; Chen et al., 2015; Lazaro et al., 2017). Several *in vivo* studies have described tBHQ cytoprotective actions under pathological conditions. Indeed, tBHQ treatment suppresses ischemia and reperfusion injury in brain (Shih et al., 2005; Hou et al., 2018; Xu et al., 2018) and kidney (Guerrero-Beltrán et al., 2012). Neuroprotective actions have been also reported in experimental models of traumatic brain injury (Saykally et al., 2012; Chandran et al., 2017), Alzheimer’s disease (Akhter et al., 2011), and neonatal hypoxic-ischemic brain damage (Zhang et al., 2018). Moreover, tBHQ ameliorates overload-induced cardiac dysfunction by suppressing apoptosis and promoting autophagy (Lin et al., 2014; Zhang et al., 2015), and also improves angiogenesis and heart function in a model of myocardial infarction (Zhou et al., 2017). tBHQ results from butylated hydroxyanlisole biotransformation, and readily auto-oxidizes to an electrophilic metabolite, tert-butylbenzoquinone (tBQ). It has been described that tBQ chemically modifies KEAP1 protein by covalent binding through its reactive cysteines (Cys23, Cys151, Cys226, and Cys368). Due to the resultant profound conformational change in KEAP1, Nrf2 escapes from KEAP1-mediated proteasomal degradation and is able to trigger the antioxidant response (Abiko et al., 2011).

Because uncontrolled inflammation, oxidative stress, and defective autophagy all concur in the pathogenesis of diabetic micro- and macrovascular complications (Schrijvers et al., 2011; Tabas et al., 2015), we hypothesize that targeting Nrf2 in the diabetic milieu could lead to a concerted upregulation of cytoprotective pathways in the vasculature to mitigate atherosclerosis progression. This study aims to explore *in vivo* and *in vitro* the effects and underlying mechanisms of the Nrf2 activator tBHQ, on preventing diabetes-associated atherosclerosis. To that end, *in vivo* studies were performed in streptozotocin (STZ)-induced diabetic apolipoprotein E-deficient (apoE^-/-^) mice, an insulin-deficient model that combines hyperglycemia and hyperlipidemia and develops accelerated vascular injury with similarities to human atherosclerosis (Hsueh et al., 2007; Chew et al., 2010). And finally, *in vivo* results were further mechanistically characterized *in vitro* in vascular cell cultures.

## Materials and Methods

### Diabetes Model and Treatments

The housing and care of animals and all the procedures performed in this study were strictly in accordance with the Directive 2010/63/EU of the European Parliament and were approved by the Institutional Animal Care and Use Committee of IIS-Fundacion Jimenez Diaz (Reference No. PROEX 116/16).

#### Study I: Atherogenesis Model (**Figure 1A**)

**FIGURE 1:**
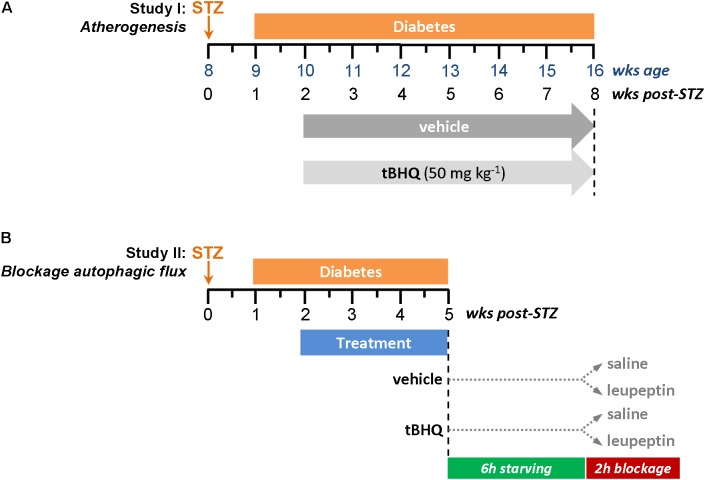
Experimental protocols for diabetes induction in apoE^-/-^ mice. **(A)** Study I: Atherogenesis model. **(B)** Study II: *In vivo* blockage of autophagic flux model.

Experimental diabetes was induced in 8-week old male apoE^-/-^ mice (Jackson Laboratory, Bar Harbor, ME, United States) by intraperitoneal injection of STZ (125 mg/kg/day in 10 mmol/L citrate buffer, pH 4.5; S0130, Sigma-Aldrich, St. Louis, MO, United States) once a day for two consecutive days (Recio et al., 2014; Lazaro et al., 2015). Animals were maintained on standard diet and monitored every 2–3 days (always at the same time of day) for body weight and non-fasting blood glucose. Mice with overt diabetes (glucose >19.4 mmol/L) were randomized to receive 6 weeks of treatment with tBHQ (50 mg/kg/day; 112941, Sigma-Aldrich, St. Louis, MO, United States; *n* = 15) or vehicle (5% ethanol in saline; *n* = 15), via intraperitoneal injection every second day. Age-matched non-diabetic mice (tBHQ and vehicle; *n* = 6 each group) were used as controls. At the study endpoint, 16 h-fasted mice were anesthetized (100 mg/kg ketamine and 15 mg/kg xylazine), saline-perfused and euthanized. Blood, urine, and tissue (aorta and liver) samples were collected. Serum levels of glucose, cholesterol (total, HDL, and LDL), triglycerides, and transaminases were measured by automated methods. Serum and urine concentrations of 8-hydroxy-2′-deoxyguanosine (8-OHdG) were assessed by ELISA (SKT-120, StressMarq Biosciences, Inc., Victoria, BC, Canada); total antioxidant capacity by colorimetric assay (STA-360, OxiSelect TAC Assay Kit, Cell Biolabs, Inc., San Diego, CA, United States).

#### Study II: *In Vivo* Blockage of Autophagic Flux (**Figure 1B**)

Diabetic apoE^-/-^ mice were treated with tBHQ and vehicle (*n* = 6/group) for 3 weeks (same protocol as described for Study I). Circadian and nutritional regulation of autophagy was considered in experimental design for autophagic flux measurement (Haspel et al., 2011; Ma et al., 2011). We injected intraperitoneally a single dose of leupeptin 20 mg/kg b.w. (BP2662, Fisher Scientific, Waltham, MA, United States) or saline into 6 h starved mice during dark cycle, and harvested tissues (aorta and liver) 2 h later.

### Atherosclerotic Lesion Analysis

To analyze plaque area and composition, the upper aortic root was embedded in Tissue-Tek OCT Compound (Sakura Finetek Europe, Alphen aan den Rijn, Netherlands) and cryosectioned. Atherosclerotic lesion area (μm^2^) and neutral lipid content were quantified in serial 8 μm aortic sections (covering about 1,000 μm from valve leaflets) after oil-red-O/hematoxylin staining and averages were calculated from 2 to 3 sections. The average foam cell size was calculated by counting the number of nuclei (hematoxylin staining) in lipid-rich areas (oil-red-O positive staining) of sections from 10 randomly selected mice per group. Total macrophages (CD68; ab53444, Abcam, Cambridge, United Kingdom), macrophage phenotypes [M1 marker arginase (ARG) 2, sc-20151; M2 marker ARG1, sc-18354; Santa Cruz Biotechnology, Inc., Santa Cruz, CA, United States], VSMCs (α-smooth muscle actin; ab5694, Abcam) and HMOX1 protein (ADI-SPA-895, Enzo Life Sciences, Farmingdale, NY, United States) were detected by immunoperoxidase (HRP-conjugated secondary antibodies: anti-rabbit, 711-035-152; anti-mouse, 715-035-150; anti-rat, 712-035-150; Jackson ImmunoResearch, West Grove, PA, United States) or immunofluorescence (Alexa Fluor 488/568 antibodies: anti-rabbit, A-11011; anti-goat, A-11055; Thermo Fisher Scientific, Waltham, MA, United States). Activated Nrf2 was detected by *in situ* Southwestern histochemistry using digoxigenin-labeled probe (Mallavia et al., 2013; Lazaro et al., 2015, 2017). Intracellular superoxide anion (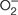) in atherosclerotic lesions was assessed by microscopy using the 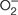-sensitive fluorescent dye dihydroethidium (DHE; D1168, Life Technologies, Carlsbad, CA, United States), followed by nuclear counterstain (DAPI; D9542, Sigma-Aldrich, St. Louis, MO, United States) (Mallavia et al., 2013). As negative control, adjacent sections were treated with polyethylene glycol-superoxide dismutase (PEG-SOD 500 U/mL; Sigma, S9549) for 2 h before DHE to determine the specificity of the fluorescence signal. All the histological evaluations were conducted in a blinded fashion. Positive staining was quantified in at least two sections per mice using Image Pro-Plus (Media Cybernetics, Bethesda, MD, United States) and expressed as percentage or number of positive cells per lesion area.

### Cell Cultures

Vascular smooth muscle cell (VSMC) were isolated from mouse aorta by enzymatic digestion with collagenase type II (C6885, Sigma-Aldrich, St. Louis, MO, United States), cultured in DMEM (D6546, Sigma-Aldrich, St. Louis, MO, United States) supplemented with 10% FBS (F7524, Sigma-Aldrich, St. Louis, MO, United States), and used between 2nd and 8th passages (Recio et al., 2014; Lazaro et al., 2015). Murine bone marrow-derived macrophages were obtained after 7 days in DMEM containing 10% FBS and 10% L929 cell-conditioned medium as a source of macrophage colony stimulating factor (Recio et al., 2014; Lazaro et al., 2015). All culture media were supplemented with 2 mmol/L L-glutamine, 100 U/mL penicillin and 100 μg/mL streptomycin (G7513 and P0781, Sigma-Aldrich, St. Louis, MO, United States). Cell viability was assessed by the 1-(4,5-dimethylthiazol2-yl)-3,5-diphenylformazan thiazolyl blue formazan (MTT; M5655, Sigma-Aldrich, St. Louis, MO, United States) method. Quiescent cells pretreated with tBHQ (5–25 μmol/L for 1–3 h) were exposed to the combination of inflammatory cytokines [10^2^ units/mL interleukin (IL)-6 plus 10^3^ units/mL interferon-γ (IFNγ); 216-16 and 315-05, PeproTech, Rocky Hill, NJ, United States], then processed and analyzed for gene and protein expressions. For autophagy experiments, cells in serum-deprived (0% FBS) or serum-supplemented (10% FBS) medium were treated with tBHQ for 90 min prior to incubation with autophagy inhibitors (100 μmol/L leupeptin plus 20 mmol/L NH_4_Cl; L2884 and A9434, Sigma-Aldrich, St. Louis, MO, United States) for additional 2 h (Bejarano et al., 2014).

### mRNA Expression Analysis

Total RNA from mouse tissues and cultured cells was extracted with TRIzol (Life Technologies). The resulting total RNA was quantified using a Nanodrop ND-1000 Spectrophotometer (NanoDrop Technologies, Wilmington, DE, United States). For each RNA sample, 1.5 μg of total RNA was reversely transcribed into cDNA using High-Capacity cDNA Reverse Transcription Kit (Applied Biosystems, Foster City, CA, United States). Real-time PCR was performed using a 7,500 Fast Real-Time PCR system (Applied Biosystems, Foster City, CA, United States) with TaqMan or SYBR Green Gene Expression detection assays. Expression levels of target genes were normalized to 18S housekeeping gene. The relative expression was determined using the formula 2^-ΔCt^.

### Protein Expression Analysis

For total protein extraction, mouse tissues and cells were homogenized in 0.25 M sucrose buffer containing 0.2 mmol/L Na_3_VO_4_, 10 mmol/L NaF, 0.2 mmol/L PMSF, and protease inhibitor cocktail (P8340, Sigma-Aldrich, St. Louis, MO, United States). Nuclear fractions were obtained by cellular homogenization in 10 mmol/L HEPES (pH 7.8), 15 mmol/L KCl, 2 mmol/L MgCl_2_, 1 mmol/L EDTA, 1 mmol/L dithiothreitol, 1 mmol/L PMSF, and protease inhibitors. Proteins were resolved on SDS-PAGE gels, transferred and immunoblotted for Nrf2 (sc-722, Santa Cruz Biotechnology, Beverly, MA, United States; orb6544, Biorbyt, Ltd., Cambridge, United Kingdom), HMOX1, microtubule-associated protein 1 light chain 3 (MAP1LC3B; 2,775, Cell Signaling), SQSTM1/p62 (sc-28359, Santa Cruz Biotechnology) and beclin-1 (BECN1; sc-11427, Santa Cruz Biotechnology), using histone H3 (4499, Cell Signaling), β-actin (sc-47778, Santa Cruz Biotechnology) and α-tubulin (T5168, Sigma-Aldrich) as loading controls. Peroxidase-conjugated secondary antibodies (anti-rabbit 711-035-152, anti-mouse 715-035-150, Jackson ImmunoResearch) were used for chemiluminescence detection. Blots were quantified using Quantity One software (Bio-Rad Laboratories, Hercules, CA, United States). For monitoring autophagic flux blockage *in vivo* (Study II) and *in vitro*, tissue and cell lysates were immunoblotted for MAP1LC3B or SQSTM1/p62 and the autophagic flux rate was determined as the amount of accumulated proteins (normalized to loading control) in the presence of lysosomal protease inhibitors vs. protease inhibitor-free conditions (Bejarano et al., 2014; Klionsky et al., 2016).

### Statistical Analysis

Results are presented as individual data points and mean ± SEM of duplicate/triplicate determinations from separate animals and cell experiments. Statistical analyses were performed using Prism 5 (GraphPad Software, Inc., La Joya, CA, United States). Differences across groups were considered significant at *P* < 0.05 using either non-parametric Mann–Whitney *U*-test, or one-way ANOVA with *post hoc* Bonferroni pairwise comparison test when appropriate.

## Results

### *In Vivo* and *in Vitro* Induction of Nrf2 Activity by tBHQ

**FIGURE 2:**
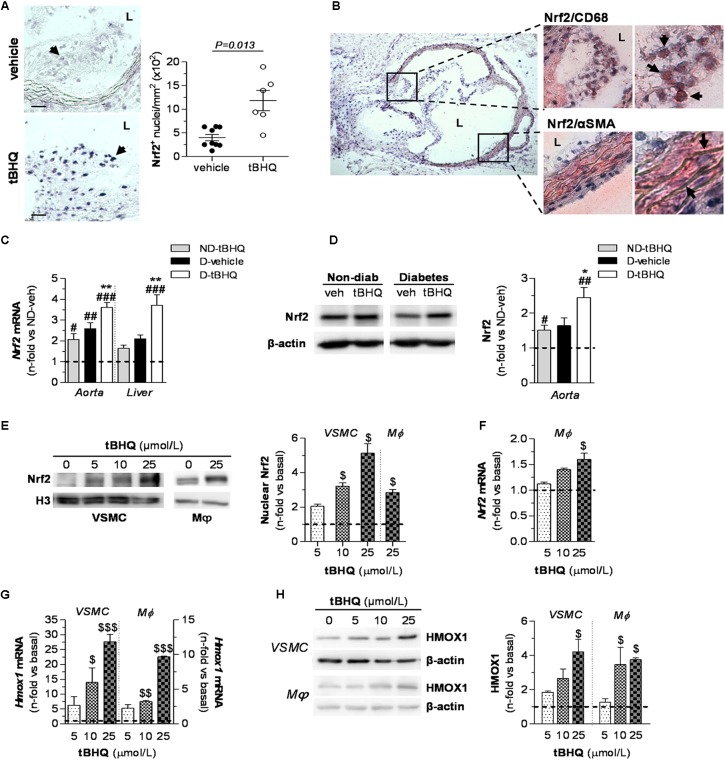
Nrf2 activation by tBHQ *in vivo* and *in vitro*. **(A,B)** *In situ* detection of activated Nrf2 by Southwestern histochemistry in aorta of diabetic apoE^-/-^ mice (2 sections per mice) after 6 weeks of treatment with either vehicle (*n* = 9) or tBHQ (*n* = 6). **(A)** Representative images (magnification ×200, scale bars: 40 μm) and quantification of positive cells per lesion area are shown. Arrows indicate Nrf2 positive cells (blue–purple). Results are presented as individual data points and mean ± SEM (*P*-value as indicated). **(B)** Representative micrographs (magnification ×40) and higher-magnifications (×200 and ×400) of the boxed regions showing colocalization of Nrf2 with macrophages (CD68) and VSMC (α-SMA). Arrows indicate double positive staining (blue–purple, Nrf2; red-brown, cell type marker). L, lumen. **(C)** Real-time PCR analysis of *Nrf2* gene expression in aorta and liver from non-diabetic and diabetic mice treated with vehicle or tBHQ. The mRNA values were normalized to 18S rRNA. **(D)** Western blot analysis of Nrf2 levels in aortic protein extracts (β-actin as loading control). Shown are representative blots and the summary of normalized densitometric quantification. Data in **(C,D)** are presented as mean ± SEM [non-diabetic (ND)-vehicle, *n* = 4; ND-tBHQ, *n* = 4; diabetic **(D)**-vehicle, *n* = 6; D-tBHQ, *n* = 9] of the relative (fold change) expression over control group (ND-vehicle; indicated by horizontal dashed lines). ^#^*P* < 0.05, ^##^*P* < 0.01, and ^###^*P* < 0.001 vs. ND-vehicle; ^∗^*P* < 0.05 and ^∗∗^*P* < 0.01 vs. D-vehicle. **(E)** Representative Western blot images and quantification of Nrf2 in nuclear extracts from cultured VSMC and macrophages (Mϕ) incubated with increasing concentrations of tBHQ (0–25 μmol/L) for 3 h. Histone H3 was used as nuclear loading control. Real-time PCR analysis of *Nrf2* **(F)** and *Hmox1* **(G)** gene expression at 6 h of treatment with tBHQ. Data are normalized to 18S. **(H)** Western blot analysis of HMOX1 (β-actin as loading control) in total cell lysates from VSMC and macrophages incubated for 24 h with tBHQ. Representative immunoblots and summary of normalized densitometry values are shown. Data in **(E–H)** are expressed as fold increases over basal conditions (indicated by horizontal dashed lines) and are the mean ± SEM of *n* = 3 independent experiments analyzed in duplicate. ^$^*P* < 0.05, ^$$^*P* < 0.01, and ^$$$^*P* < 0.001 vs. basal.

To explore *in vivo* whether Nrf2 induction protects against development of diabetes-driven atherosclerosis, we set up an experimental model of accelerated vascular injury alike to human atherosclerotic lesions, resulting from the combination of hyperglycemia and hyperlipidemia (Hsueh et al., 2007; Chew et al., 2010; Recio et al., 2014; Lazaro et al., 2015). In this study, streptozotocin-induced diabetic apoE^-/-^ mice were treated with either vehicle or tBHQ for 6 weeks. *In situ* southwestern histochemistry revealed a 2.2-fold increase in the number of Nrf2-activated cells within atherosclerotic lesions of tBHQ-treated mice (**Figure 2A**). Activated Nrf2 colocalized with both macrophages and VSMC (**Figure 2B**) which are main cellular constituents of atherosclerotic lesions with an active role in atherogenesis. In addition, tBHQ treatment induced Nrf2 expression at both mRNA and protein levels in the aorta of non-diabetic and diabetic mice, as showed by real-time PCR (**Figure 2C**) and Western blot (**Figure 2D**). The induction of Nrf2 gene was also confirmed in liver tissue from tBHQ-treated mice (**Figure 2C**). According to these *in vivo* observations, we next tested *in vitro* the ability of tBHQ to bolster intrinsic Nrf2 pathway in cultures of VSMC and macrophages. Western blot analysis revealed increased levels of Nrf2 protein in nuclear fractions from both VSMC and macrophages incubated with tBHQ (**Figure 2E**). Real-time PCR analysis confirmed a dose-dependent induction of Nrf2 gene by tBHQ in primary macrophages (**Figure 2F**). Consistently, tBHQ dose-dependently induced the gene and protein expression of HMOX1, which is a Nrf2-downstream target gene, in both cell types (**Figures 2G,H**).

### Atheroprotective Effect of tBHQ Treatment in Non-diabetic and Diabetic Mice

**FIGURE 3:**
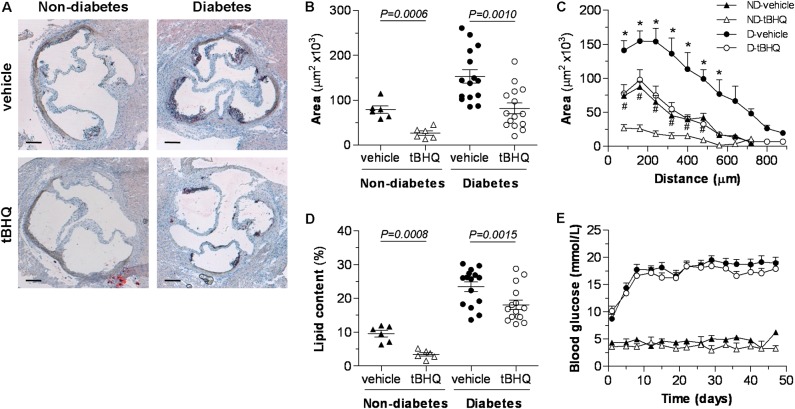
tBHQ treatment lessens the development of atherosclerosis in mice. **(A)** Representative images (magnification ×40, scale bars: 200 μm) of oil-red-O/hematoxylin staining in aortic root sections from non-diabetic and diabetic mice treated with either vehicle or tBHQ. **(B)** Average of individual maximal lesion area in each group (calculated from 2 to 3 sections per mice). **(C)** Quantification of the extent of atherosclerotic lesions within the aorta (9–11 sections per mice). **(D)** Quantification of intraplaque lipid content (% oil-red-O per lesion area, calculated from 2 to 3 sections per mice in individual maximal lesion area). **(E)** Evolution of mean blood glucose values in non-fasted mice along the 6-week follow-up. Results are expressed as individual data points and mean ± SEM [non-diabetic (ND)-vehicle, *n* = 6; ND-tBHQ, *n* = 6; diabetic (D)-vehicle, *n* = 15; D-tBHQ, *n* = 15]. ^∗^*P* < 0.05 vs. D-vehicle; ^#^*P* < 0.05 vs. ND-vehicle.

Quantitative assessment of aortic root sections after oil-red-O/hematoxylin staining evidenced the role of diabetes as a driving force in the early progression of atherosclerosis (twofold increase in lesion area in diabetic vs. non-diabetic mice; *P* = 0.0004) (**Figures 3A,B**). Interestingly, tBHQ treatment significantly reduced the size (**Figure 3B**) and extension (**Figure 3C**) of atherosclerotic lesions both in non-diabetic and diabetic mice compared to their respective age-matched vehicle controls (% of decrease: non-diabetic, 66 ± 6; diabetic, 46 ± 8). In addition, plaques from tBHQ-treated mice displayed lower neutral lipid content (% of decrease: non-diabetic, 64 ± 5; diabetic, 28 ± 5; **Figure 3D**). Throughout the study, non-fasting blood glucose levels remained similar between vehicle and tBHQ in both non-diabetic and diabetic groups (**Figure 3E**). At the end of the study, there were no significant changes in body weights and serum lipid levels (**Table 1**) after tBHQ treatment. Serum transaminase activities kept similar between vehicle and tBHQ groups, indicating preserved liver function (**Table 1**).

**Table 1:** Biochemical data of apoE^-/-^ mice after 6 weeks of treatment.

	Non-diabetic		Diabetic	
	Vehicle	tBHQ	Vehicle	tBHQ
		
	(*n* = 6)	(*n* = 6)	(*n* = 15)	(*n* = 15)
Body weight change (final–initial) (g)	3.2 ± 0.0	0.0 ± 3.0	-2.8 ± 1.0	-3.0 ± 1.0
Total cholesterol (mg/dL)	330 ± 19	263 ± 18	852 ± 47###	935 ± 69###
LDL cholesterol (mg/dL)	293 ± 18	227 ± 13	819 ± 42###	901 ± 66###
HDL cholesterol (mg/dL)	15.3 ± 0.6	15.5 ± 0.8	10.3 ± 0.7##	11.2 ± 0.7#
Triglycerides (mg/dL)	111 ± 10	124 ± 16	109 ± 15	112 ± 15
Aspartate aminotransferase (units/L)	169 ± 4	169 ± 25	188 ± 21	184 ± 14
Alanine aminotransferase (units/L)	51 ± 7	52 ± 7	86 ± 11	88 ± 8
Serum total antioxidant capacity (mmol/L)	0.34 ± 0.04	0.62 ± 0.06##	0.19 ± 0.02	0.42 ± 0.03*
Urine total antioxidant capacity (mmol/L)	1.5 ± 0.1	2.4 ± 0.1###	1.2 ± 0.1	2.0 ± 0.1**,#

*Data are mean ± SEM. *^#^P* < 0.05, *^##^P* < 0.01, and *^###^P* < 0.001 vs. non-diabetic vehicle group. ^∗^*P* < 0.05 and ^∗∗^*P* < 0.01 vs. diabetic vehicle group*.

### Impact of Nrf2 Activation on Inflammation and Redox Balance

**FIGURE 4:**
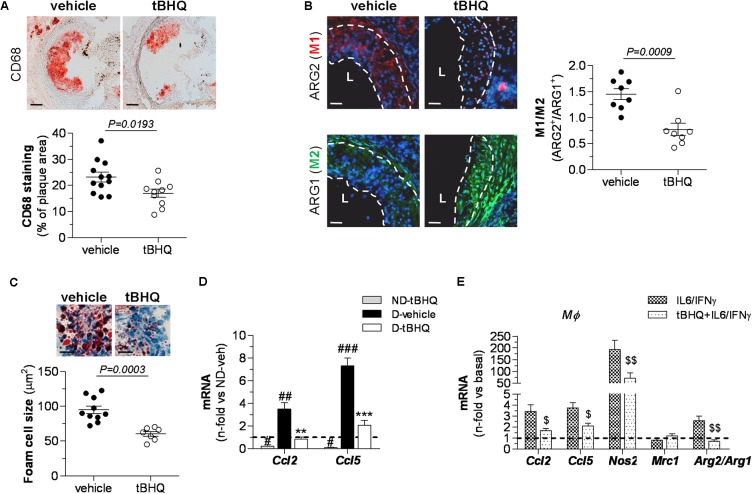
Reduced inflammation in atherosclerotic plaques of tBHQ-treated mice. **(A)** Immunoperoxidase detection of total macrophages (magnification ×100, scale bars: 80 μm) and quantitative analysis of CD68+ cells in aortic root sections of diabetic mice (vehicle, *n* = 12; tBHQ, *n* = 10; 2 sections per mice). **(B)** Immunofluorescence analysis of macrophage phenotypes (*red*, ARG2 (M1); *green*, ARG1 (M2); *blue*, DAPI (nuclear staining); magnification ×200; scale bars: 20 μm; L, lumen) and quantification of M1/M2 ratio in aorta of diabetic mouse (vehicle, *n* = 8; tBHQ, *n* = 8; 2 sections per mice). **(C)** Representative images (magnification x200, scale bars: 40 μm) of foam cell-rich areas in atherosclerotic lesions of diabetic mice (vehicle, *n* = 10, and tBHQ, *n* = 7; 2–3 sections per mice) and quantitative assessment of foam cell size. **(D)** Real-time PCR analysis of inflammatory markers [chemokines *Ccl2* and *Ccl5*) in aortic tissue from non-diabetic (ND)-vehicle (*n* = 4), ND-tBHQ (*n* = 4), diabetic (D)-vehicle (*n* = 10), and D-tBHQ (*n* = 10) mice]. Data normalized by 18S are expressed as fold increases over control group (ND-vehicle, represented by horizontal dashed line). **(E)** Real-time PCR analysis of *Ccl2* and *Ccl5*, macrophage phenotype markers *Nos2* (M1), *Mrc1* (M2), and *Arg2/Arg1* (ratio M1/M2) in primary macrophages pretreated with tBHQ (25 μmol/L, 90 min) or vehicle prior to stimulation with cytokines (IL-6 10^2^ units/mL plus IFNγ 10^3^ units/mL) for 6 h. Data normalized by 18S are expressed as fold increases over basal condition (vehicle-treated cells, represented by horizontal dashed line; *n* = 6 independent experiments). Results are expressed as individual data points and mean ± SEM. ^∗∗^*P* < 0.01 and ^∗∗∗^*P* < 0.001 vs. D-vehicle; ^#^*P* < 0.05, ^##^*P* < 0.01, and ^###^*P* < 0.001 vs. ND-vehicle; ^$^*P* < 0.05 and ^$$^*P* < 0.01 vs. cytokine stimulation.

Histological assessment of diabetic mouse aorta showed that tBHQ treatment limited the accumulation of macrophages (CD68 positive staining) within atherosclerotic plaques (**Figure 4A**) and also altered the phenotypic distribution of macrophages in atheroma by reducing M1 marker (ARG2) and increasing M2 marker (ARG1) (**Figure 4B**). Furthermore, calculation of average foam cell size in atherosclerotic plaques demonstrated that lesional macrophages in tBHQ-treated mice were smaller than in vehicle group (**Figure 4C**), indicating a reduced lipid accumulation in these cells. Likewise, real-time PCR analysis in aortic samples evidenced that tBHQ significantly reduced the chemokine gene pattern both in non-diabetic and diabetic mice (**Figure 4D**), thus confirming its anti-inflammatory function.

Next, we explored *in vitro* the Nrf2-dependent anti-inflammatory effects of tBHQ in macrophages stimulated with cytokines (IL-6 plus IFNγ), in an attempt to mimic the inflammatory environment of the atheroma plaque. As shown in **Figure 4E**, tBHQ prevented cytokine-induced expression of chemokines C-C motif chemokine ligand (CCL) 2 and CCL5. In addition, tBHQ primed macrophages toward the anti-inflammatory M2 phenotype, as evidenced by decreased expression of inducible nitric oxide synthase (NOS2, marker of pro-inflammatory M1 phenotype), modest increase in mannose receptor C-type 1 (MRC1, M2 marker), and lower ratio of ARG isoforms [ARG2 (M1) to ARG1 (M2)].

**FIGURE 5:**
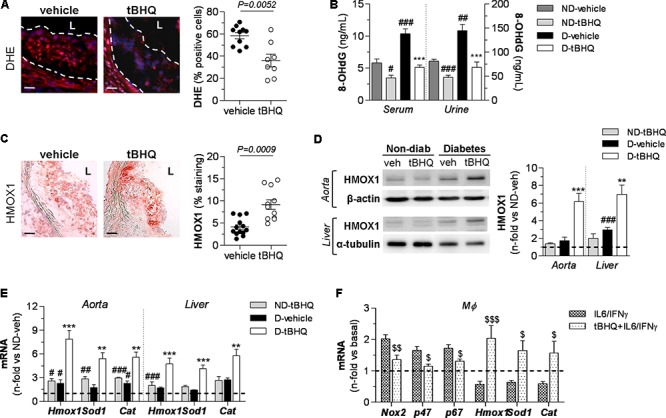
tBHQ treatment attenuates oxidative stress and promotes antioxidant response in mice. **(A)** *In situ* detection of 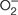 production by DHE fluorescent probe labeling in aortic sections of diabetic mice (vehicle, *n* = 10; tBHQ, *n* = 8; 2 sections per mice). Representative images (magnification x200, scale bars: 40 μm; L, lumen) and quantification of percentage of positive DHE red-cells vs. total DAPI blue-nuclei. **(B)** Concentrations of 8-OHdG in serum and urine samples from non-diabetic (ND)-vehicle (*n* = 4), ND-tBHQ (*n* = 6), diabetic (D)-vehicle (*n* = 3) and D-tBHQ (*n* = 6) mice. **(C)** Immunoperoxidase images (magnification ×100, scale bars: 80 μm; L, lumen) of HMOX1 protein in aortic sections of diabetic mice (vehicle, *n* = 13; tBHQ, *n* = 11; 2 sections per mice) and summary of quantification. **(D)** Western blot analysis of HMOX1 in aorta and liver extracts from ND-vehicle (*n* = 4), ND-tBHQ (*n* = 4), D-vehicle (*n* = 11) and D-tBHQ (*n* = 11) mice (β-actin and α-tubulin as loading controls). Shown are representative blots and the summary of normalized densitometry expressed as fold increases over control group (ND-vehicle, represented by horizontal dashed line). **(E)** Real-time PCR analysis of antioxidant defense genes (*Hmox1*, *Sod1*, and *Cat*) in aorta and liver from ND-vehicle (*n* = 4), ND-tBHQ (*n* = 4), D-vehicle (*n* = 12) and D-tBHQ (*n* = 12) groups. Data normalized by 18S are expressed as fold increases over control group (ND-vehicle, represented by horizontal dashed line). **(F)** Real-time PCR analysis of Nox subunits and antioxidant enzymes in primary macrophages pretreated with tBHQ (25 μmol/L, 90 min) prior to stimulation with cytokines (IL-6 10^2^ units/mL plus IFNγ 10^3^ units/mL) for 6 h. Data are normalized to 18S and expressed as fold increases over basal condition (vehicle-treated cells, represented by horizontal dashed line; *n* = 6 independent experiments). Results are expressed as individual data points and mean ± SEM. ^∗∗^*P* < 0.01 and ^∗∗∗^*P* < 0.001 vs. D-vehicle; ^#^*P* < 0.05, ^##^*P* < 0.01, and ^###^*P* < 0.001 vs. ND-vehicle; ^$^*P* < 0.05, ^$$^*P* < 0.01, and ^$$$^*P* < 0.001 vs. cytokine stimulation.

To assess the impact of tBHQ on redox balance, we analyzed oxidative stress markers in atherosclerotic plaques. *In situ* detection of 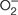 production by PEG-SOD-inhibitable DHE fluorescence in aortic sections revealed that tBHQ treatment markedly reduced the number of DHE^+^ cells in atherosclerotic plaques compared to vehicle-treated group (**Figure 5A**). Concomitantly, tBHQ almost reversed the increase of serum and urinary levels of 8-OHdG (a marker of oxidative DNA damage) to control levels (**Figure 5B**). Next, we examined the tBHQ effectiveness on antioxidant defense by assessing Nrf2-driven antioxidant genes not only in aorta but also in hepatic tissue, due to the important role of liver in atherogenesis as an essential hub of lipid metabolism. Immunodetection of HMOX1 protein revealed higher expression levels in atherosclerotic plaques (**Figure 5C**) and also in protein extracts from aortic and hepatic tissues (**Figure 5D**) of tBHQ-treated mice. Real-time PCR analysis also showed upregulated gene expression of the antioxidant enzymes HMOX1, SOD1, and CAT in both diabetic and non-diabetic mice receiving tBHQ when compared with respective vehicle-treated groups (**Figure 5E**). A parallel analysis of the total antioxidant status revealed that tBHQ recovered the impaired capacity of serum and urine to resist oxidation (**Table 1**). Complementary *in vitro* studies in primary macrophages confirmed that tBHQ altered the macrophage redox balance, with reduced gene expression of Nox components (membrane-associated Nox2 protein and cytosolic p47^phox^ and p67^phox^ regulatory subunits), alongside increased expression of antioxidant enzymes HMOX1, SOD1, and CAT (**Figure 5F**).

### Effect of tBHQ Treatment on Autophagy Mechanisms *in Vivo* and *in Vitro*

**FIGURE 6:**
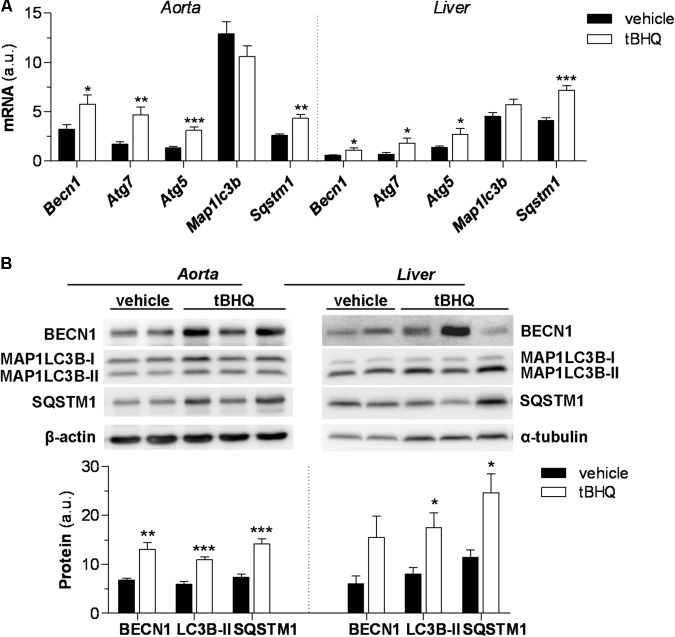
tBHQ promotes autophagy in diabetic mouse. **(A)** Real-time PCR analysis of autophagy genes in aortic and hepatic tissues from diabetic mice treated with either vehicle (*n* = 15) or tBHQ (*n* = 15). Values normalized by 18S are expressed as arbitrary units. **(B)** Western blot analysis of autophagy markers BECN1, MAP1LC3B-II, and SQSTM1/p62 in aorta and liver of diabetic mice (D-vehicle, *n* = 13; D-tBHQ, *n* = 14). Values normalized by loading controls (β-actin and α-tubulin) are expressed as arbitrary units. Results are expressed as mean ± SEM. ^∗^*P* < 0.05, ^∗∗^*P* < 0.01, and ^∗∗∗^*P* < 0.001 vs. D-vehicle.

To investigate the involvement of autophagy in Nrf2-mediated atheroprotection we analyzed the expression of genes involved in key steps of autophagy, from initiation (BECN1), to autophagosome formation (ATG7, ATG5, and MAP1LC3B) and targeting polyubiquitinated proteins for degradation (SQSTM1/p62). Real-time PCR analysis in diabetic mouse aorta showed that tBHQ treatment significantly increased the mRNA expression of autophagy genes, except for MAP1LC3B (**Figure 6A**). Western blot further confirmed upregulated levels of BECN1 and SQSTM1/p62 proteins, and conversion of MAP1LC3B-I to MAP1LC3B-II form in tBHQ-treated mice (**Figure 6B**). Similar results were observed in liver (**Figures 6A,B**), thus corroborating the beneficial systemic effects of tBHQ.

**FIGURE 7:**
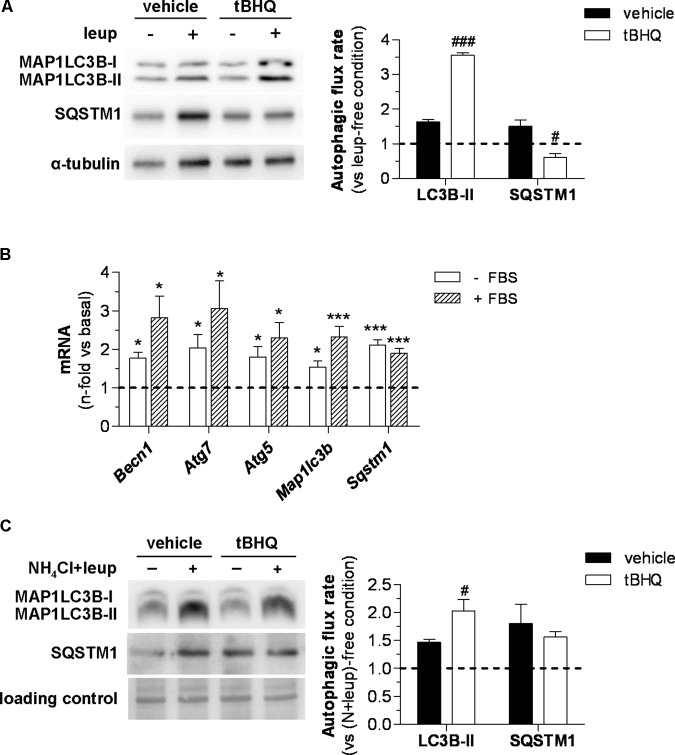
*In vivo* and *in vitro* effect of tBHQ on autophagic activity. **(A)** *In vivo* 2 h blockage of autophagic flux in 6 h starved diabetic mice treated with either vehicle or tBHQ for 3 weeks by intraperitoneal injection of leupeptin (vehicle-group, *n* = 3; tBHQ, *n* = 3) or saline (vehicle-group, *n* = 3; tBHQ, *n* = 3). Shown are representative blots of MAP1LC3B-II, SQSTM1/p62 and α-tubulin (loading control) and quantification of autophagic flux rate in aortic tissue. **(B)** Real-time PCR analysis of autophagy genes in VSMC treated with tBHQ 25 μmol/L for 6 h in serum-deprived (-FBS) or serum-supplemented (+FBS) conditions. Data normalized by 18S are expressed as fold increase over respective basal conditions (*n* = 9 independent experiments). **(C)** *In vitro* blockage of autophagic flux in starved VSMC pretreated with either tBHQ (25 μmol/L, 90 min) or vehicle prior to the addition of lysosomal inhibitors (20 mmol/L NH_4_Cl plus 100 μmol/L leupeptin; N-leup) for 2 h. Shown are representative blots and quantification of autophagic flux rate in VSMC (*n* = 7 independent experiments). The rate of autophagic flux is expressed as lysosomal protease inhibitor (leup or N-leup) induced protein accumulation (MAP1LC3B-II or SQSTM1/p62) vs. respective lysosomal protease inhibitor-free condition. Results are expressed as mean ± SEM. ^∗^*P* < 0.05 and ^∗∗∗^*P* < 0.001 vs. basal; ^#^*P* < 0.05 and ^###^*P* < 0.001 vs. respective lysosomal protease inhibitor-free condition.

In addition to the steady-state changes of autophagy markers, we also studied the effect of tBHQ on autophagic flux by assessing MAP1LC3B-II levels in the presence or absence of lysosomal protease inhibitors. In these experiments, blockage of lysosomal proteolysis *in vivo* was assessed by intraperitoneal injection of leupeptin in 6 h starved diabetic mice. Under these conditions, Western blot analysis revealed that tBHQ increases autophagic flux in the vasculature, as evidenced by higher accumulation of MAP1LC3B-II protein in tBHQ-treated mice compared with vehicle group (**Figure 7A**). However, no accumulation of autophagic adaptor SQSTM1/p62 was found in the aortic tissue upon tBHQ treatment (**Figure 7A**). Finally, we investigated the *in vitro* effects of tBHQ on autophagy machinery. Real-time PCR analysis showed upregulated BECN1, ATG5/7, MAP1LC3B, and SQSTM1/p62 gene expression by tBHQ in VSMC under both pro-autophagic (serum-deprived medium) and basal (serum-supplemented medium) conditions (**Figure 7B**). Moreover, increased autophagic flux rate by tBHQ was underscored by the higher accumulation of MAP1LC3B-II protein, but not SQSTM1/p62, in VSMC incubated with lysosomal inhibitors (**Figure 7C**).

## Discussion

The present study provides mechanistic evidence of the atheroprotective effects of Nrf2 pathway beyond its role in commanding the antioxidant response. We report that Nrf2 activation by tBHQ counteracts diabetes-accelerated atherosclerosis through a multiple cytoprotective action based on the attenuation of vascular inflammation and oxidative stress, and bolstering autophagy.

Previous reports demonstrated that myeloid-specific Nrf2 deletion in mice aggravates early and advanced stages of atherosclerosis (Ruotsalainen et al., 2013), and controversially, total gene deficiency protects against atherogenesis by affecting both systemic and local mechanisms (Barajas et al., 2011; Freigang et al., 2011). Our work indicates that administration of tBHQ in diabetic mice further activated Nrf2 in macrophages and VSMC, two main cellular components of atherosclerotic lesions with a key role in atherogenesis (Moore and Tabas, 2011; Razani et al., 2012; Salabei and Hill, 2013). Associated with Nrf2 induction in diabetic mouse aorta, tBHQ treatment reduced the area, extension, lipid content and inflammatory milieu of atherosclerotic plaques, independently of changes in blood glycemia and lipids. Our results are in agreement with the previously reported effect of tBHQ in ischemic stroke (Shih et al., 2005) and cardiac dysfunction (Zhang et al., 2015) and support the use of Nrf2 activators as potential therapy for diabetic complications (Xue et al., 2008; Jiang et al., 2010; Tan and de Haan, 2014; Tan et al., 2014).

Macrophage accumulation in the subendothelial space contributes to atherogenesis by promoting inflammatory response and plaque instability (Moore and Tabas, 2011). Our results showed that tBHQ treatment impaired the accumulation of macrophages within atherosclerotic lesions, but also affected the macrophage inflammatory state by favoring the alternatively activated M2 phenotype. Previous studies have determined a linkage of M2 macrophages with fatty acid oxidation (Vats et al., 2006), a process regulated by Nrf2 (Dinkova-Kostova and Abramov, 2015). In this sense, tBHQ might modulate macrophage capacity to catabolize extravased modified lipoproteins, leading to smaller-sized foam cells. Additionally, tBHQ treatment attenuated the aortic expression of CCL2 and CCL5, two prototypical chemokines of fundamental importance in the atherosclerotic process (Zernecke et al., 2008; van der Vorst et al., 2015). Either gene deficiency or pharmacological inhibitions of CCL2, CCL5, and their cognate receptors have been demonstrated to reduce lesion size and macrophage infiltration and to promote plaque stability in mice. In vascular cells and leukocytes, CCL2 and CCL5 expression by many atherogenic stimuli is transcriptionally regulated by nuclear factor κB (NF-κB) (Monaco and Paleolog, 2004). Evidence indicates a functional crosstalk between Nrf2 and NF-κB pathways. In macrophages, Nrf2 opposes transcriptional upregulation of proinflammatory cytokine genes (Kobayashi et al., 2016) and its absence exacerbates NF-κB activity and promotes atherogenesis (Ruotsalainen et al., 2013). Conversely, NF-κB represses Nrf2 by a physical interaction with KEAP1 (Yu et al., 2011). We have reported opposite regulation of NF-κB and Nrf2 by heat shock protein 90 inhibitor in diabetic atherosclerosis (Lazaro et al., 2017). In consonance, our results reinforce Nrf2 as direct regulator of inflammation, and help reconsider the concept of Nrf2-mediated anti-inflammation as an off-target effect of ROS suppression.

Hyperglycemia-induced ROS generation by Nox family promotes cellular damage and contributes to development and progression of vascular diabetic complications (Giacco and Brownlee, 2010; Jiang et al., 2011; Rask-Madsen and King, 2013). In atherosclerotic lesions, macrophages and VSMC contribute to Nox-derived ROS production (Vendrov et al., 2007), which is exacerbated in the absence of Nrf2 (Kong et al., 2010; Ashino et al., 2013). In line with this, our study demonstrates that tBHQ treatment attenuated oxidative stress both locally (reduced 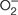 production in aorta) and systemically (lower circulating levels of oxidative DNA stress marker 8-OHdG) in diabetic mice. Moreover, tBHQ suppressed the cytokine-induced expression of Nox subunits in macrophages. It is well-known that diabetes itself promotes antioxidant defense in aortas, as an adaptative response to overcome injury due to the chronic exposure to hyperglycemia-driven oxidative stress (Giacco and Brownlee, 2010; Rask-Madsen and King, 2013). Remarkably, we observed that tBHQ treatment further boosted Nrf2 system for the expression of antioxidant enzymes (HMOX1, SOD1, and CAT). Besides the positive effects on the aortic tissue, we also showed that tBHQ reinforced antioxidant machinery in liver and restored systemic total antioxidant capacity in diabetic mice.

Autophagy is activated in response to several stressors such as starvation, lipids, ROS and cytokines to maintain cellular integrity, but chronic insults can result in autophagic impairment and loss of vascular cellular homeostasis thus contributing to atherogenesis (Schrijvers et al., 2011; Salabei and Hill, 2013). Autophagy related gene deficiency in macrophages promotes atherosclerosis by triggering inflammasome hyperactivation, Nox-mediated oxidative stress, apoptosis, defective efferocytosis and necrosis (Liao et al., 2012; Razani et al., 2012), whereas VSMC-specific deletion promotes a phenotype switch with attenuated proliferative capacity, ensuing migration and premature senescence (Salabei and Hill, 2013; Grootaert et al., 2015). Moreover, defective lipid management due to a blunted autophagy impedes cholesterol efflux and contributes to foam cell formation (Singh et al., 2009; Koga et al., 2010). In line with this, the atheroprotection found in diabetic mice after tBHQ treatment can be linked to the restoration of cellular autophagy and the subsequent attenuation of macrophage infiltration, intracellular lipid content and foam cell formation.

Nrf2-KEAP1-ARE axis and autophagy are linked by SQSTM1/p62 protein. Indeed, direct interaction between SQSTM1/p62 and KEAP1 favors its autophagy-mediated degradation with subsequent activation of Nrf2-driven genes (Komatsu et al., 2010; Lau et al., 2010). Nrf2 upregulates SQSTM1/p62 and ATG5 genes thus creating a positive feedback loop (Jain et al., 2010; Pajares et al., 2016). In agreement, our results showed that Nrf2 activation by tBHQ upregulated gene and protein expression of components of the autophagy machinery in diabetic mice. Remarkably, tBHQ promoted autophagic activity, as measured by MAP1LC3B-II accumulation, the gold standard method to monitor autophagic activity (Klionsky et al., 2016). The data *in vivo* (aorta and liver of diabetic mice) were consistent with the data *in vitro* (primary VSMC), and together they demonstrate structural and functional modulation of autophagy by Nrf2 activation. It has previously been reported that SQSTM1/p62 protein plays a pivotal role between autophagy and proteasome degradation pathways in a MAP1LC3B-II-independent manner (Itakura and Mizushima, 2011; Liebl and Hoppe, 2016). Therefore, and according to previous reports in other systems (Jain et al., 2010; Xu et al., 2016), it is likely that the protective role of tBHQ involves an increase of SQSTM1/p62 and an activation in proteasomal activity, and be related with a switch in the shuttling of SQSTM1/p62 observed in diabetic mouse aorta and cultured VSMC.

Collectively, this study proposes pharmacological Nrf2 activation as a useful therapeutic strategy to restrain diabetes-driven atherosclerosis, by attenuating oxidative stress and inflammation, and bolstering antioxidant defense and autophagy.

## Author Contributions

IL designed experiments, researched and analyzed data, and wrote the manuscript. LL-S researched, analyzed or interpreted data, and revised the manuscript. SB, AO, CR, AM, and LJ-C performed *in vivo* studies and revised the manuscript. JE critically revised the manuscript for important intellectual content. JM-M designed experiments and critically revised the manuscript. CG-G designed the study, analyzed data, and wrote the manuscript.

## Conflict of Interest Statement

The authors declare that the research was conducted in the absence of any commercial or financial relationships that could be construed as a potential conflict of interest.

## Abbreviations

apoE^-/-^: apolipoprotein E-deficient
ARE: antioxidant response element
ARG: arginase
ATG5: autophagy-related gene 5
ATG7: autophagy-related gene 7
BECN1: beclin 1
CAT: catalase
CCL C-C motif: chemokine ligand
DHE: dihydroethidium
DMEM: Dulbecco’s modified Eagle medium
FBS: fetal bovine serum
HMOX: heme oxygenase 1
IL-6: interleukin 6
IFNγ: interferon γ
KEAP1: Kelch-like ECH-associated protein 1
MAP1LC3B: microtubule-associated protein 1A/1B light chain B
MRC1: mannose receptor C-type 1
NF-κB: nuclear factor κB
NOS2: inducible nitric oxide synthase
Nox: NADPH oxidase
Nrf2: nuclear factor (erythroid-derived 2)-like 2
superoxide anion
8-OHdG: 8-hydroxy-2′-deoxyguanosine
ROS: reactive oxygen species
SMA: smooth muscle actin
SOD1: superoxide dismutase 1
SQSTM1/p62: sequestosome 1
STZ: streptozotocin
tBHQ: tert-butyl hydroquinone
tBQ: tert-butylbenzoquinone
VSMC: vascular smooth muscle cell.
